# Understanding the impact of socioeconomic differences in colorectal cancer survival: potential gain in life-years

**DOI:** 10.1038/s41416-019-0455-0

**Published:** 2019-05-01

**Authors:** Elisavet Syriopoulou, Eva Morris, Paul J. Finan, Paul C. Lambert, Mark J. Rutherford

**Affiliations:** 1 Biostatistics Research Group, Department of Health Sciences, University of Leicester, University Road, LE1 7RH Leicester, UK; 2 Cancer Epidemiology Group, Institute of Medical Research at St James’s and Institute of Data Analytics, University of Leeds, Worsley Building, Leeds, LS2 9JT UK; 3 Department of Medical Epidemiology and Biostatistics, Karolinska Institutet, 171 77 Stockholm, Sweden

**Keywords:** Epidemiology, Outcomes research

## Abstract

**Background:**

Colorectal cancer prognosis varies substantially with socioeconomic status. We investigated differences in life expectancy between socioeconomic groups and estimated the potential gain in life-years if cancer-related survival differences could be eliminated.

**Methods:**

This population-based study included 470,000 individuals diagnosed with colon and rectal cancers between 1998 and 2013 in England. Using flexible parametric survival models, we obtained a range of life expectancy measures by deprivation status. The number of life-years that could be gained if differences in cancer-related survival between the least and most deprived groups were removed was also estimated.

**Results:**

We observed up to 10% points differences in 5-year relative survival between the least and most deprived. If these differences had been eliminated for colon and rectal cancers diagnosed in 2013 then almost 8231 and 7295 life-years would have been gained respectively. This results for instance in more than 1-year gain for each colon cancer male patient in the most deprived group on average. Cancer-related differences are more profound earlier on, as conditioning on 1-year survival the main reason for socioeconomic differences were factors other than cancer.

**Conclusion:**

This study highlights the importance of policies to eliminate socioeconomic differences in cancer survival as in this way many life-years could be gained.

## Background

Although overall colorectal cancer survival rates are improving there is substantial variation by deprivation, age and to some extent sex. Previous studies investigating inequalities among socioeconomic groups showed that the most deprived group have a significantly worse prognosis after diagnosis.^1,2^ The deprivation gap is unlikely to be explained entirely by differences in tumour characteristics.^3^ In a randomised clinical trial, in which equal treatment was provided, the deprivation inequalities were reduced suggesting that other factors related to the healthcare system, such as access to treatment, might account for the variation seen.^4^

To understand the impact of eliminating inequalities on patients’ whole lifespan, loss in life expectancy measures can be used. Loss in life expectancy due to a cancer diagnosis is defined as the reduction in life expectancy following a cancer diagnosis and is given as the difference of the life expectancy in the general population that is free of the cancer of interest and the life expectancy in the population of cancer patients.^5^ In comparison with the commonly reported measure of relative survival that estimates survival in a hypothetical world, where the only possible cause of death is the cancer of interest, loss in life expectancy is an intuitive measure that makes communication of cancer survival easier.^6^ It is a measure of great interest in public health as it can be used to quantify the disease burden in society and to address various research questions such as the impact of cancer diagnosis on life expectancy among different populations.^7^

In addition to absolute estimates of loss in life expectancy other measures can also be estimated. The proportion of life lost provides a measure for the impact of cancer that depends less on age and accounts for the fact that younger patients have more years to lose. Conditional measures can also be obtained to provide an updated estimate of prognosis for patients who survived a certain number of years. Moreover, total life-years lost based on a specific year’s number of diagnoses is useful for estimating the population impact of cancer.^8^

This study estimated the impact of colon and rectal cancers on patients’ life expectancy across socioeconomic groups in England using a range of reporting measures. It also quantified the number of life-years that could be gained if differences in cancer-related survival between the least and most deprived groups were removed. Proportional and conditional measures are also provided.

## Methods

### Data resources

This retrospective population-based study includes all patients diagnosed with colon and rectal cancers from 1998 until 2013 in England. Data were obtained from National Cancer Registration and Analysis Service within Public Health England. International Classification of Diseases 10 was used to identify colon cancer (C18) and rectal cancer (C19, C20). Each patient was assigned to one out of five deprivation groups. The information on deprivation status is a weighted average based on national quantiles of the index of multiple deprivation (IMD) 2010 score based on the patients’ residence at diagnosis and is not an individual specific measure.^9,10^

### Statistical methods

A flexible parametric relative survival model was fitted separately for colon and rectal cancers as well as males and females. Flexible parametric models use restricted cubic splines to model the baseline excess hazard.^11,12^ In our analysis, 5 degrees of freedom were considered to model the log cumulative baseline excess hazard allowing for 6 knots for the splines. Knots are points at which the splines join. Age at diagnosis was included in the model as a continuous variable and a non-linear effect was allowed by using restricted cubic splines with 3 degrees of freedom. As it is common in population-based cancer studies to have a larger effect of age on excess mortality earlier in follow-up, time-dependent effects (non-proportional excess hazards) for age, were also included in the models assuming 3 degrees of freedom. The models also included the main and time-dependent effect of deprivation status (3 degrees of freedom for the time-dependent effect) and an interaction between age and deprivation status to allow a differential effect of age across the deprivation groups.

A period window from the beginning of year 2007 to the end of year 2013 was used, allowing only for the follow-up time during this window to be included in the analysis. In period analysis, patients that were diagnosed further back in the follow-up and were still under follow-up during the period window contribute to long-term survival estimates whereas survival of patients that were diagnosed recently is used for short-term survival. In this way, better estimates for newly diagnosed patients are obtained.^13,14^

Relative survival is defined as the all-cause survival divided by the expected survival in the general population that is free of the cancer of interest and has similar characteristics with the cancer population. Expected survival rates were incorporated in the models by using population mortality life tables stratified by sex, age at diagnosis, calendar year, and deprivation status.^15^ For each model, estimates of relative survival, loss in life expectancy, proportion of life lost and conditional loss in loss in life expectancy given that patients survived 1 year after their diagnosis by deprivation group were obtained. The proportion of life lost was calculated as the loss in life expectancy divided by the expected life expectancy. The conditional measure of loss in life expectancy was used to assess whether cancer differences remain after 1 year of survival. The total years lost due to cancer diagnosis across deprivation groups in England in 2013, the most recent year in the available data, was obtained by multiplying the number of patients diagnosed with colon or rectal cancer in 2013 with the average loss in life expectancy. The average loss in life expectancy was calculated as a weighted average of the age-specific estimates within each deprivation group.

The impact of eliminating inequalities in cancer-related survival across socioeconomic groups was then estimated. First, the above loss in life expectancy measures were estimated once more using the relative survival estimates of the least deprived group for all the other deprivation groups. Subsequently, the difference between estimates where each group was allowed to have their own relative survival and estimates obtained under the scenario that a group’s relative survival was the same as the relative survival of the least deprived group were produced. By using the relative survival of the least deprived group it was then possible to quantify the potential gain in life-years if cancer-related differences in survival could be removed.

## Results

The analysis included more than 300,000 and 170,000 patients diagnosed with colon and rectal cancer respectively. Female colon cancer patients were diagnosed at a slightly older age in comparison with male patients, with an average age of 71 years for males and 73 years for women. A similar pattern was observed for rectal cancer with the average age being 69 and 71 years old for male and female patients respectively (Table 1).

**Table 1:** Number and mean age of patients diagnosed with colon and rectal cancer from 1998 until 2013 in England by deprivation status for males and females

Cancer type	Gender	Deprivation group	Number of patients	Mean age (years)
Colon	Males	Least deprived	32,079	70.66
		2	34,187	71.23
		3	32,618	71.34
		4	29,593	71.14
		Most deprived	25,855	70.20
	Females	Least deprived	28,687	71.98
		2	32,140	73.00
		3	31,885	73.32
		4	29,186	73.33
		Most deprived	24,167	72.39
Rectal	Males	Least deprived	21,125	68.57
		2	22,542	69.19
		3	22,411	69.43
		4	20,858	69.31
		Most deprived	19,029	68.62
	Females	Least deprived	12,995	70.08
		2	14,500	70.96
		3	14,403	71.52
		4	13,414	71.58
		Most deprived	11,484	70.73

Table 2 shows the 5-year relative survival estimates of males diagnosed with colon cancer at specific ages by deprivation group. Differences in relative survival between the least and the most deprived groups remained above 5.5% points and in favour of the most affluent group for all ages considered. The highest difference was observed for patients diagnosed at the age of 60, where 5-year relative survival was 65.87% for the least and 56.35% for the most deprived patients. Similarly, large differences in relative survival were observed for females with colon cancer as well as rectal cancer (Supplementary Tables 1, 2, and 3). Table 2 and Supplementary Tables 1, 2, and 3 also show that relative survival remains at similar levels for ages 50, 60, and 70 years old but a larger decrease is observed for those diagnosed at 80 years old.

**Table 2:** Years lost by deprivation group if male colon cancer patients diagnosed at the ages of 50, 60, 70, 80 years old had (i) their own relative survival and (ii) the same relative survival as the least deprived group

					RS = As least deprived group		
Deprivation group	5-year RS	Mean years w/o cancer	Mean years with cancer	Prop (%)	Mean years with cancer	Prop (%)	Years gained
Age-at-diagnosis: 50							
Least deprived	65.21	33.58	19.92	40.67	19.92	40.67	0.00
2	62.08	32.30	18.51	42.69	19.24	40.43	0.73
3	61.08	31.11	17.58	43.49	18.59	40.23	1.01
4	60.30	29.39	16.95	42.32	17.66	39.92	0.70
Most deprived	55.87	27.30	14.15	48.17	16.51	39.53	2.36
Age-at-diagnosis: 60							
Least deprived	65.87	24.43	14.89	39.04	14.89	39.04	0.00
2	63.71	23.30	13.94	40.17	14.28	38.69	0.34
3	61.73	22.35	12.98	41.93	13.76	38.43	0.78
4	59.20	20.97	11.98	42.86	13.00	38.02	1.01
Most deprived	56.35	19.42	10.38	46.55	12.13	37.56	1.75
Age-at-diagnosis: 70							
Least deprived	63.02	16.08	9.81	38.99	9.81	38.99	0.00
2	61.26	15.12	9.11	39.78	9.30	38.49	0.20
3	60.03	14.49	8.59	40.73	8.95	38.22	0.36
4	56.90	13.56	7.78	42.60	8.44	37.77	0.65
Most deprived	54.74	12.66	6.94	45.15	7.93	37.36	0.99
Age-at-diagnosis: 80							
Least deprived	50.96	9.35	5.04	46.06	5.04	46.06	0.00
2	50.26	8.62	4.66	46.00	4.71	45.34	0.06
3	50.45	8.35	4.53	45.72	4.58	45.14	0.05
4	47.96	7.92	4.16	47.46	4.38	44.77	0.21
Most deprived	45.32	7.69	3.81	50.40	4.25	44.67	0.44

Loss in life expectancy after a colon cancer diagnosis varies by age at diagnosis and deprivation group (Table 2). Larger differences are observed across socioeconomic groups. For example, 60-year-old males in the general population that are free of colon cancer are expected to live on average for 24.43 years if they belong to the least deprived group and 19.42 years if they belong in the most deprived group. A cancer diagnosis at that age however may result in a decrease in life expectancy and is estimated that the least deprived will have a life expectancy of 14.89 years and the equivalent life expectancy for the most deprived is 10.38 years. The proportion of life lost is significantly higher for the most deprived patient. Loss in life expectancy caused after diagnosis results in 39.04% loss in the expected remaining life of the least deprived patient and 46.55% for the most deprived. However, by eliminating the differences in cancer-related survival and therefore forcing the relative survival of the most deprived to the be the same as for the least deprived patients, the proportion of life lost for the most deprived decreases to 37.56%. This is even smaller than the equivalent proportion of the most affluent patients. Such high differences in loss in life expectancy are observed also for females and rectal cancer with females are losing more years than men (Supplementary Tables 1, 2, and 3).

**Fig. 1:**
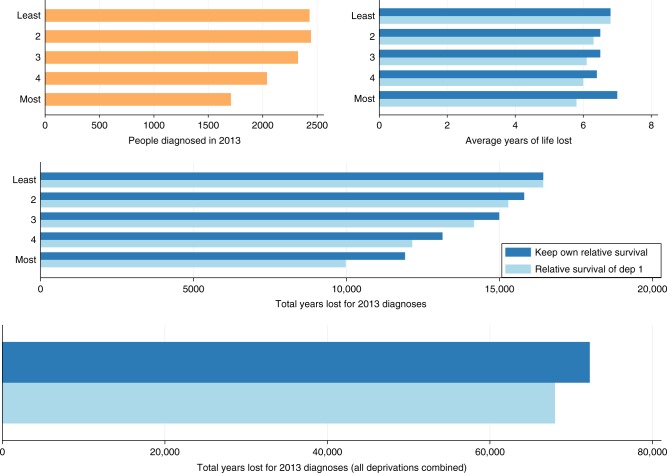
Number of male patients diagnosed with colon cancer in 2013, the average life years lost and the total years lost by deprivation group (1 for the least deprived and 5 for the most deprived patients) as well as total years lost for all deprivation groups combined (plot in the bottom) under two scenarios (i) if each group had their own relative survival (dark blue bar) and (ii) if each groups had the relative survival of the least deprived group (light blue bar)

**Fig. 2:**
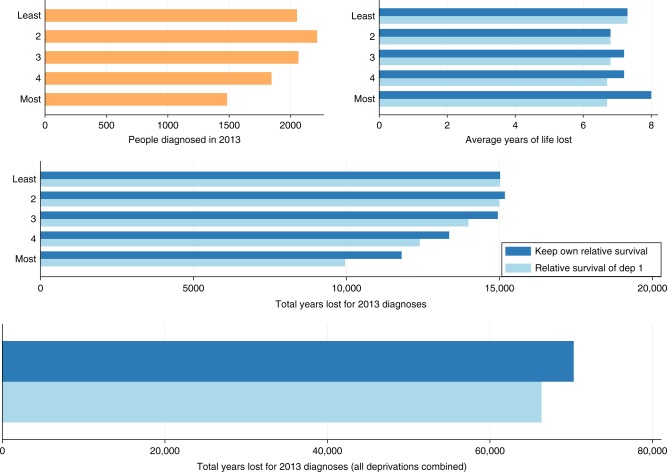
Number of female patients diagnosed with colon cancer in 2013, the average life years lost and the total years lost by deprivation group (1 for the least deprived and 5 for the most deprived patients) as well as total years lost for all deprivation groups combined (plot in the bottom) under two scenarios (i) if each group had their own relative survival (dark blue bar) and (ii) if each groups had the relative survival of the least deprived group (light blue bar)

The impact of cancer on a population level is estimated in Table 3. For males with colon cancer, there were 6.76 and 6.98 years lost on average for the least and most deprived patients respectively. Based on the number of patients diagnosed with the disease in 2013, this accounts for 16,431 life-years lost among the least deprived and 11,914 life-years lost among the most deprived patients. However, if cancer-related differences in survival were eliminated and the relative survival of the most affluent group was applied to the most deprived then the least deprived would lose 9977 life-years i.e., 1937 life-years could be gained for a cohort size and composition of those diagnosed in 2013. The most deprived females could also gain 1854 years. For rectal cancer, the equivalent gain for the most deprived would be 1739 years for males and 1157 years for females. By eliminating difference in all deprivation groups, there would be 4270 and 3961 life-years gained in total for males and females respectively (Figs. 1 and 2). Similarly, for rectal cancer there would be 4348 life-years and 2947 life-years gained for males and females respectively. As it is shown in Supplementary Figs. 1 and 2, rectal cancer results in a smaller number of total life-years lost in comparison with colon cancer due to the smaller number of patients diagnosed with rectal cancer in 2013, even though the average loss in life expectancy is slightly higher for rectal cancer.

**Table 3:** Total years lost, for colon and cancer patients, based on 2013 diagnosis if patients had (i) their own relative survival or (ii) the same relative survival as the least deprived group, by gender and deprivation group

				RS = As least deprived group		
Deprivation group	Group size in 2013	Mean life years lost	Total life years lost	Mean life years lost	Total life years lost	Life years gained
Colon cancer						
Males						
Least deprived	2430	6.76	16,431	6.76	16,431	0
2	2443	6.47	15,808	6.26	15,289	519
3	2324	6.45	14,992	6.10	14,169	823
4	2039	6.44	13,138	5.96	12,147	991
Most deprived	1708	6.98	11,914	5.84	9977	1937
Females						
Least deprived	2053	7.32	15,022	7.32	15,022	0
2	2217	6.85	15,178	6.76	14,996	182
3	2065	7.24	14,949	6.77	13,986	963
4	1845	7.24	13,357	6.72	12,395	962
Most deprived	1483	7.96	11,804	6.71	9950	1854
Rectal cancer						
Males						
Least deprived	1464	7.10	10,384	7.10	10,384	0
2	1499	6.90	10,412	6.50	9790	622
3	1395	6.90	9654	6.30	8745	909
4	1245	6.90	8553	6.00	7475	1078
Most deprived	1158	7.40	8534	5.90	6795	1739
Females						
Least deprived	790	7.30	5761	7.30	5761	0
2	816	7.00	5714	6.80	5567	147
3	843	7.40	6265	6.40	5409	856
4	779	7.40	5784	6.40	4997	787
Most deprived	642	8.10	5208	6.30	4051	1157

Table 4 shows the partitioning of differences in life expectancy between the least and most deprived groups to that due to cancer or due to other causes. For example, for two female patients diagnosed with colon cancer at age 70 years old, one of whom belong to the least and the other to the most deprived group, a difference of 3.15 years in life expectancy is observed. If we could eliminate the cancer-related differences, then a difference of 1.66 years would still be observed (Supplementary Table 1). Thus, 47.4% of the differences in prognosis of the two groups are due to cancer and the remaining 52.6% are due to other causes. A similar pattern is observed for all ages. As time since diagnosis increases however the proportions differ significantly. Given that the previous 70-year-old patients survive 1 year after their diagnosis, the differences we observed between them would mainly be explained by causes different to their cancer. Specifically, cancer accounts only for 28.2% of the differences whereas other causes account for 71.8% of the observed differences. For older female patients, a slightly higher proportion of differences could be attributed to causes unrelated to cancer. Findings were similar for male patients. For rectal cancer we observed the same pattern and after conditioning on 1 year of survival cancer was explaining a smaller proportion of the socioeconomic differences in comparison with the unconditional estimates.

**Table 4:** Proportion of differences in life expectancy, between the least and most deprived groups, which can be explained by differences in either cancer differences or other cause differences by age at diagnosis for male and female colon cancer patients. Measures are given both based on the beginning of follow up after diagnosis as well as conditioning on 1-year survival

Gender	Age at diagnosis	Unconditional		Conditioning on 1-year survival	
		Proportion due to cancer	Proportion due to other	Proportion due to cancer	Proportion due to other
Colon cancer					
Males	50	40.90	59.10	26.74	73.26
	60	38.68	61.32	24.11	75.89
	70	34.45	65.55	16.27	83.73
	80	35.76	64.25	3.08	96.92
Females	50	41.06	58.94	27.70	72.30
	60	46.47	53.53	31.81	68.19
	70	47.42	52.58	28.24	71.76
	80	50.69	49.31	18.32	81.68
Rectal cancer					
Males	50	43.58	56.42	31.88	68.12
	60	44.34	55.66	32.79	67.21
	70	37.65	62.35	21.11	78.89
	80	44.06	55.94	15.26	84.74
Females	50	54.32	45.68	46.86	53.14
	60	54.40	45.60	46.18	53.82
	70	48.01	51.99	35.38	64.62
	80	46.59	53.41	24.10	75.90

Finally, Supplementary Fig. 3, shows the loss in life expectancy for a female patient diagnosed with colon cancer at the age of 70. For patients at that age who will survive their cancer for 1 year, the loss in life expectancy is estimated to be lower and differences between the least and most deprived patients of this age are also decreasing. The reduction in loss in life expectancy of the patient can partly be explained by the fact that there are less years to lose to begin with as the patient has already survived for 1 year but are mostly explained by patients having survived through the year following diagnosis, where the excess mortality is high.

## Discussion

This study has provided a detailed summary on the impact of colorectal cancer on patients’ life expectancy by socioeconomic status using a data resource on all patients diagnosed in England. Socioeconomic differences were quantified using measures of loss in life expectancy that assess the impact of a cancer diagnosis on the whole lifespan instead of a particular point in follow-up time after diagnosis, such as 1-year or 5-year relative survival. To better understand inequalities in prognosis, the potential gain in life-years by eliminating differences between socioeconomic groups was further explored. Removing cancer-related inequalities would result in substantial gain in life-years and our results support the importance of policies to target the most affected group and remove socioeconomic inequalities. Updated estimates conditioning on 1-year survival were also obtained to help inform our understanding of what drives the survival differences.

Results showed that each colon cancer male patient from the most deprived group would gain 1.14 years of life on average. The equivalent gain for females was 1.25 years and for rectal cancer was 1.5 and 1.8 years for males and females respectively. These numbers varied significantly with age at diagnosis. On a population level, eliminating the differences across all deprivation groups would result in almost 8231 and 7295 life-years gained based on the number of colon and rectal cancer diagnoses in 2013, respectively.

Age at diagnosis has a strong impact on loss in life expectancy and younger patients were found to lose noticeably more years of their remaining life as they have more years to lose to begin with. For example, a male colon cancer patient from the most deprived group that was diagnosed at the age of 50 would lose 13.15 years from his remaining life because of his diagnosis on average. However, if this patient had the same cancer-related survival as a similar patient from the least deprived group, he would have lost 10.79 years instead i.e., there would be 2.36 years gained. Potential gain in life-years for the younger patients is particularly important, as a number of studies had shown that the incidence of colon and rectal cancer is increasing in the under 50 s.^16–20^

An intervention of eliminating differences may not be straightforward in practice as many factors may account for the differences. Even though England has a universal healthcare system, there is evidence of differential treatment between socioeconomic groups.^21^ Previous studies have also suggested that stage at diagnosis may partially explain the differences.^21,22^ An evaluation of the population-based colorectal cancer screening programme that was initiated in 2006 showed a striking gradient by socioeconomic status with the uptake ranging from 35% in the most deprived to 61% in the least deprived.^23^ Another study indicated that emotional and practical barriers, which could affect screening decision making, were strongly negatively associated with education. If diagnosis in a more advance stage accounts for some of the inequalities, then action to promote informed uptake of screening could be implemented.^24^ Other possible factors that may influence survival are lifestyle, health-seeking behaviours as some people are more prone to seek medical care and comorbidities.^25–27^ Controlling for all the factors that drive inequalities can be challenging. An advantage of our study is that it focused on quantifying cancer-related differences rather than all-cause differences. All-cause differences may be the result of more complicated mechanisms that relates both to cancer and other causes. In contrast, it may be easier to control for cancer-related differences.

Our analysis, showed also that colon cancer accounts for 34–50% of the total differences observed in loss in life expectancy depending on the age at diagnosis. However, as time since diagnosis increases and, especially for the older patients, other causes account for the majority of the observed differences. When conditioning on 1-year survival, less than 32% of the observed differences are due to colon cancer-related differences and other causes dominate. Even when differences in cancer survival are removed, the all-cause survival continues to vary by deprivation group. A similar trend was observed for rectal cancer.

Loss in life expectancy is a useful measure for assessing the impact of a cancer diagnosis in the whole of remaining life and it yields real world estimates in which both cancer and other causes of death may be present. Their simple interpretation makes communication of cancer statistics to a broader audience easier. Proportional and conditional measures can also be estimated to provide different aspects of the cancer impact. To estimate the population impact, the total life-years lost can be calculated simply by choosing the number of patients diagnosed in a typical year.

Even though loss in life expectancy measures provide useful summaries of cancer, if interest lies in comparing cancer survival between different populations or countries then relative survival may be more appropriate. Relative survival accounts for different background mortalities between populations as it refers to a hypothetical world where the cancer of interest is the only possible cause of death and other causes of death are not present. In this paper, our aim was to provide estimates of the actual impact on colon and rectal cancers in England and therefore loss in life expectancy was more relevant as it incorporates other causes of death that are present in the real world.

This study has demonstrated large differences in terms of prognosis between socioeconomic groups. If these cancer-related differences could be eliminated many life-years could be gained for the most affected groups. Further investigation is now required in order to explore the underlying determinants that drive inequalities. A better understanding of mechanisms that generate inequalities is essential as it would enable the implementation of appropriate policies to eliminate them.

## Supplementary information

Supplemental material

## **Data availability:**

The data that support the findings of this study are available from Public Health England, but restrictions apply to the availability of these data, which were used under license for the current study, and so are not publicly available.
