# Supplementary material for: Understanding the impact of socioeconomic differences in colorectal cancer survival: potential gain in life-years
Source: Br J Cancer. 2019 May 1;120(11):1052–8. doi: 10.1038/s41416-019-0455-0 (PMC6738073; doi:10.1038/s41416-019-0455-0)

Supplementary material

Supplementary Table 1: Years lost by deprivation group if female colon cancer patients diagnosed at the ages of 50, 60, 70, 80 years old had i) their own relative survival and ii) the same relative survival as the least deprived group.

|  | | | | | RS = As least deprived group | | |
| --- | --- | --- | --- | --- | --- | --- | --- |
| Deprivation Group | 5-year RS | Mean Years w/o Cancer | Mean Years with Cancer | Prop (%) | Mean Years with Cancer | Prop (%) | Years Gained |
| Age-at-diagnosis: 50 | | | | | | | |
| Least Deprived | 64.49 | 36.37 | 21.82 | 40.02 | 21.82 | 40.02 | 0.00 |
| 2 | 64.69 | 35.19 | 21.37 | 39.28 | 21.16 | 39.87 | -0.21 |
| 3 | 62.01 | 34.34 | 19.79 | 42.37 | 20.68 | 39.76 | 0.90 |
| 4 | 62.55 | 33.13 | 19.53 | 41.05 | 20.01 | 39.61 | 0.48 |
| Most Deprived | 58.97 | 31.56 | 17.25 | 45.35 | 19.12 | 39.41 | 1.88 |
| Age-at-diagnosis: 60 | | | | | | | |
| Least Deprived | 64.83 | 27.06 | 16.57 | 38.75 | 16.57 | 38.75 | 0.00 |
| 2 | 64.47 | 26.00 | 15.95 | 38.63 | 15.98 | 38.53 | 0.03 |
| 3 | 62.21 | 25.26 | 14.87 | 41.12 | 15.56 | 38.39 | 0.69 |
| 4 | 61.58 | 24.27 | 14.29 | 41.13 | 15.00 | 38.20 | 0.71 |
| Most Deprived | 56.74 | 23.08 | 12.36 | 46.44 | 14.32 | 37.96 | 1.96 |
| Age-at-diagnosis: 70 | | | | | | | |
| Least Deprived | 63.57 | 18.26 | 11.38 | 37.65 | 11.38 | 37.65 | 0.00 |
| 2 | 62.33 | 17.35 | 10.68 | 38.48 | 10.87 | 37.36 | 0.19 |
| 3 | 60.52 | 16.82 | 10.04 | 40.31 | 10.56 | 37.21 | 0.52 |
| 4 | 58.77 | 16.12 | 9.42 | 41.60 | 10.16 | 37.01 | 0.74 |
| Most Deprived | 53.96 | 15.39 | 8.23 | 46.52 | 9.72 | 36.80 | 1.50 |
| Age-at-diagnosis: 80 | | | | | | | |
| Least Deprived | 55.84 | 10.75 | 6.27 | 41.72 | 6.27 | 41.72 | 0.00 |
| 2 | 54.14 | 10.05 | 5.72 | 43.11 | 5.90 | 41.34 | 0.18 |
| 3 | 52.81 | 9.81 | 5.46 | 44.34 | 5.77 | 41.24 | 0.30 |
| 4 | 50.55 | 9.47 | 5.08 | 46.41 | 5.58 | 41.08 | 0.51 |
| Most Deprived | 46.89 | 9.24 | 4.62 | 50.05 | 5.45 | 41.00 | 0.84 |

Supplementary Table 2: Years lost by deprivation group if male rectal cancer patients diagnosed at the ages of 50, 60, 70, 80 years old had i) their own relative survival and ii) the same relative survival as the least deprived group.

|  | | | | | RS = As least deprived group | | |
| --- | --- | --- | --- | --- | --- | --- | --- |
| Deprivation Group | 5-year RS | Mean Years w/o Cancer | Mean Years with Cancer | Prop (%) | Mean Years with Cancer | Prop (%) | Years Gained |
| Age-at-diagnosis: 50 | | | | | | | |
| Least Deprived | 68.94 | 33.58 | 20.51 | 38.90 | 20.51 | 38.90 | 0.00 |
| 2 | 63.89 | 32.30 | 18.23 | 43.55 | 19.83 | 38.61 | 1.59 |
| 3 | 63.02 | 31.11 | 17.62 | 43.35 | 19.17 | 38.37 | 1.55 |
| 4 | 62.01 | 29.39 | 16.76 | 42.97 | 18.22 | 37.99 | 1.46 |
| Most Deprived | 56.14 | 27.30 | 14.39 | 47.28 | 17.06 | 37.51 | 2.67 |
| Age-at-diagnosis:60 | | | | | | | |
| Least Deprived | 69.57 | 24.43 | 15.36 | 37.13 | 15.36 | 37.13 | 0.00 |
| 2 | 67.26 | 23.30 | 14.28 | 38.73 | 14.75 | 36.70 | 0.47 |
| 3 | 64.76 | 22.35 | 13.31 | 40.45 | 14.22 | 36.39 | 0.91 |
| 4 | 61.85 | 20.97 | 12.12 | 42.20 | 13.44 | 35.89 | 1.32 |
| Most Deprived | 55.77 | 19.42 | 10.33 | 46.82 | 12.56 | 35.33 | 2.23 |
| Age-at-diagnosis: 70 | | | | | | | |
| Least Deprived | 65.74 | 16.08 | 10.14 | 36.93 | 10.14 | 36.93 | 0.00 |
| 2 | 64.35 | 15.12 | 9.46 | 37.47 | 9.62 | 36.35 | 0.17 |
| 3 | 61.39 | 14.49 | 8.73 | 39.72 | 9.27 | 36.03 | 0.53 |
| 4 | 58.56 | 13.56 | 7.93 | 41.52 | 8.74 | 35.51 | 0.81 |
| Most Deprived | 54.71 | 12.66 | 7.07 | 44.16 | 8.23 | 35.04 | 1.16 |
| Age-at-diagnosis: 80 | | | | | | | |
| Least Deprived | 49.96 | 9.35 | 5.02 | 46.35 | 5.02 | 46.35 | 0.00 |
| 2 | 48.57 | 8.62 | 4.60 | 46.70 | 4.71 | 45.37 | 0.11 |
| 3 | 46.97 | 8.35 | 4.35 | 47.89 | 4.59 | 45.11 | 0.23 |
| 4 | 44.40 | 7.92 | 3.98 | 49.76 | 4.39 | 44.61 | 0.41 |
| Most Deprived | 41.41 | 7.69 | 3.68 | 52.17 | 4.27 | 44.49 | 0.59 |

Supplementary Table 3: Years lost by deprivation group if female rectal cancer patients diagnosed at the ages of 50, 60, 70, 80 years old had i) their own relative survival and ii) the same relative survival as the least deprived group.

|  | | | | | RS = As least deprived group | | |
| --- | --- | --- | --- | --- | --- | --- | --- |
| Deprivation Group | 5-year RS | Mean Years w/o Cancer | Mean Years with Cancer | Prop (%) | Mean Years with Cancer | Prop (%) | Years Gained |
| Age-at-diagnosis: 50 | | | | | | | |
| Least Deprived | 71.15 | 36.37 | 23.62 | 35.05 | 23.62 | 35.05 | 0.00 |
| 2 | 70.39 | 35.19 | 22.65 | 35.63 | 22.92 | 34.86 | 0.27 |
| 3 | 66.04 | 34.34 | 19.99 | 41.78 | 22.41 | 34.74 | 2.42 |
| 4 | 67.01 | 33.13 | 19.66 | 40.64 | 21.68 | 34.56 | 2.02 |
| Most Deprived | 61.25 | 31.56 | 17.28 | 45.24 | 20.73 | 34.33 | 3.45 |
| Age-at-diagnosis:60 | | | | | | | |
| Least Deprived | 72.96 | 27.06 | 18.51 | 31.58 | 18.51 | 31.58 | 0.00 |
| 2 | 71.50 | 26.00 | 17.45 | 32.88 | 17.85 | 31.34 | 0.40 |
| 3 | 68.37 | 25.26 | 15.87 | 37.17 | 17.38 | 31.19 | 1.51 |
| 4 | 66.98 | 24.27 | 14.91 | 38.59 | 16.75 | 30.99 | 1.85 |
| Most Deprived | 60.73 | 23.08 | 12.97 | 43.80 | 15.99 | 30.74 | 3.01 |
| Age-at-diagnosis: 70 | | | | | | | |
| Least Deprived | 68.09 | 18.26 | 12.19 | 33.23 | 12.19 | 33.23 | 0.00 |
| 2 | 67.00 | 17.35 | 11.45 | 34.03 | 11.64 | 32.93 | 0.19 |
| 3 | 63.82 | 16.82 | 10.47 | 37.74 | 11.30 | 32.77 | 0.83 |
| 4 | 63.16 | 16.12 | 9.95 | 38.31 | 10.87 | 32.56 | 0.93 |
| Most Deprived | 57.38 | 15.39 | 8.77 | 43.03 | 10.41 | 32.34 | 1.64 |
| Age-at-diagnosis:80 | | | | | | | |
| Least Deprived | 52.04 | 10.75 | 5.96 | 44.60 | 5.96 | 44.60 | 0.00 |
| 2 | 51.74 | 10.05 | 5.57 | 44.58 | 5.62 | 44.09 | 0.05 |
| 3 | 48.06 | 9.81 | 5.09 | 48.13 | 5.50 | 43.95 | 0.41 |
| 4 | 49.15 | 9.47 | 5.00 | 47.22 | 5.33 | 43.73 | 0.33 |
| Most Deprived | 44.91 | 9.24 | 4.56 | 50.67 | 5.21 | 43.62 | 0.65 |

Supplementary Figure 1. Number of male patients diagnosed with rectal cancer in 2013, the average life years lost and the total years lost by deprivation group (1 for the least deprived and 5 for the most deprived patients) as well as total years lost for all deprivation groups combined (plot in the bottom) under two scenarios i) if each group had their own relative survival (dark blue bar) and ii) if each groups had the relative survival of the least deprived group (light blue bar).


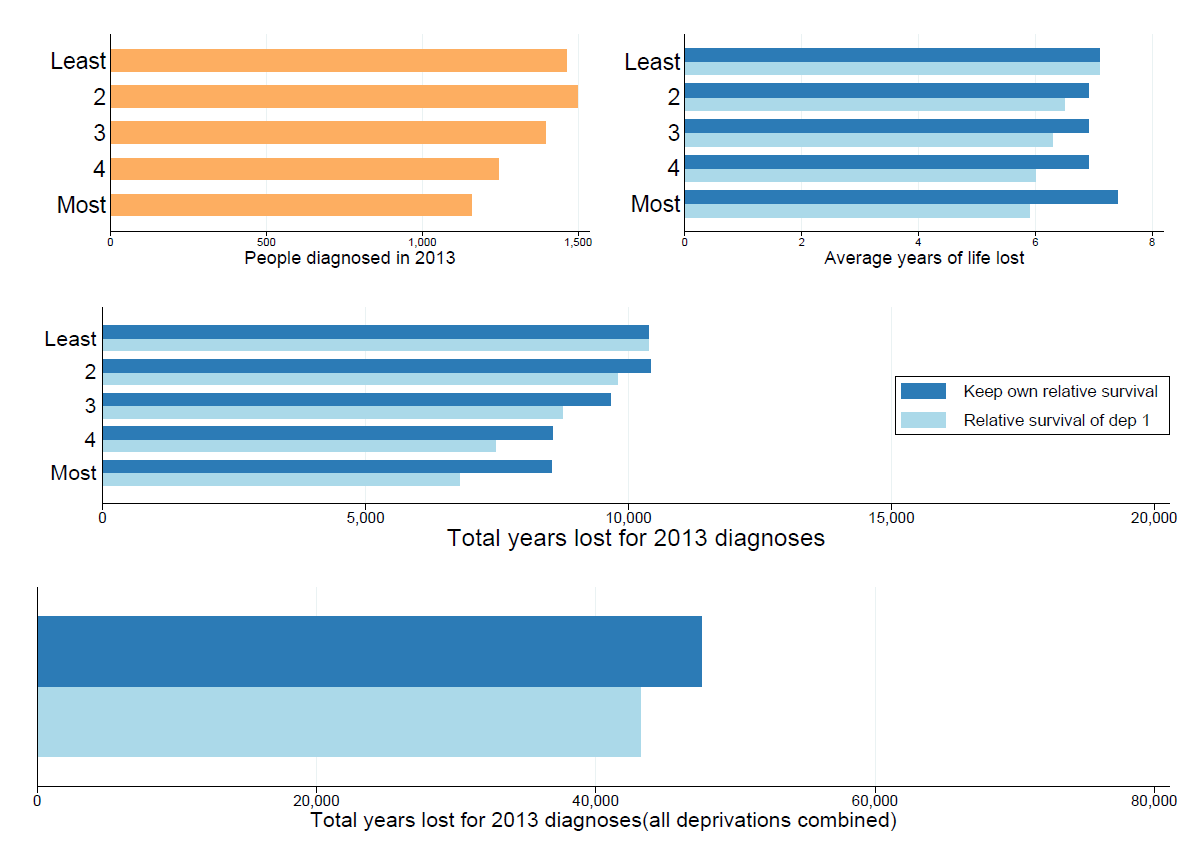


Supplementary Figure 2. Number of female patients diagnosed with rectal cancer in 2013, the average life years lost and the total years lost by deprivation group (1 for the least deprived and 5 for the most deprived patients) as well as total years lost for all deprivation groups combined (plot in the bottom) under two scenarios i) if each group had their own relative survival (dark blue bar) and ii) if each groups had the relative survival of the least deprived group (light blue bar).


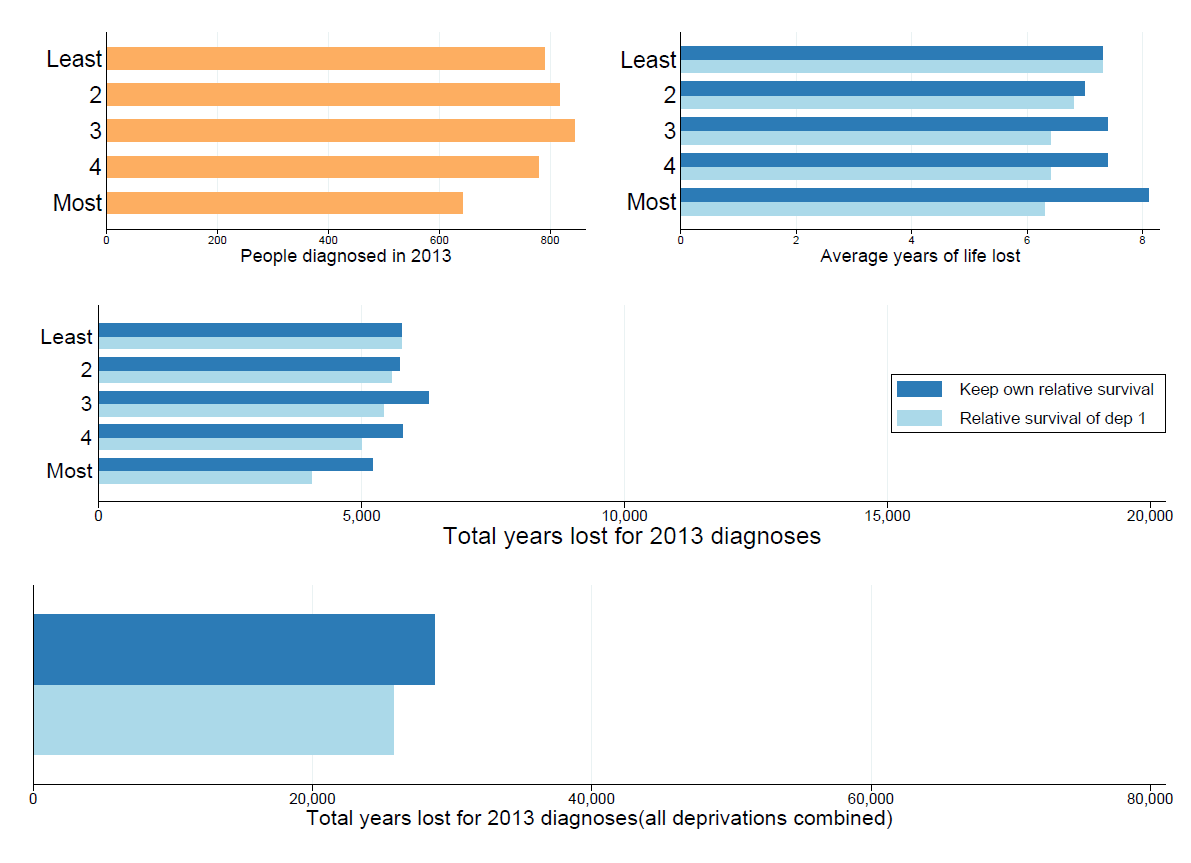


Supplementary Figure 3. Loss in life expectancy for 70-years old female patient at the most deprived group (Dep 5) if they had i) their own relative survival or ii) the same relative survival as the least deprived group. Estimates are given A) since the beginning of diagnosis and B) conditioning on 1-year of survival.

A


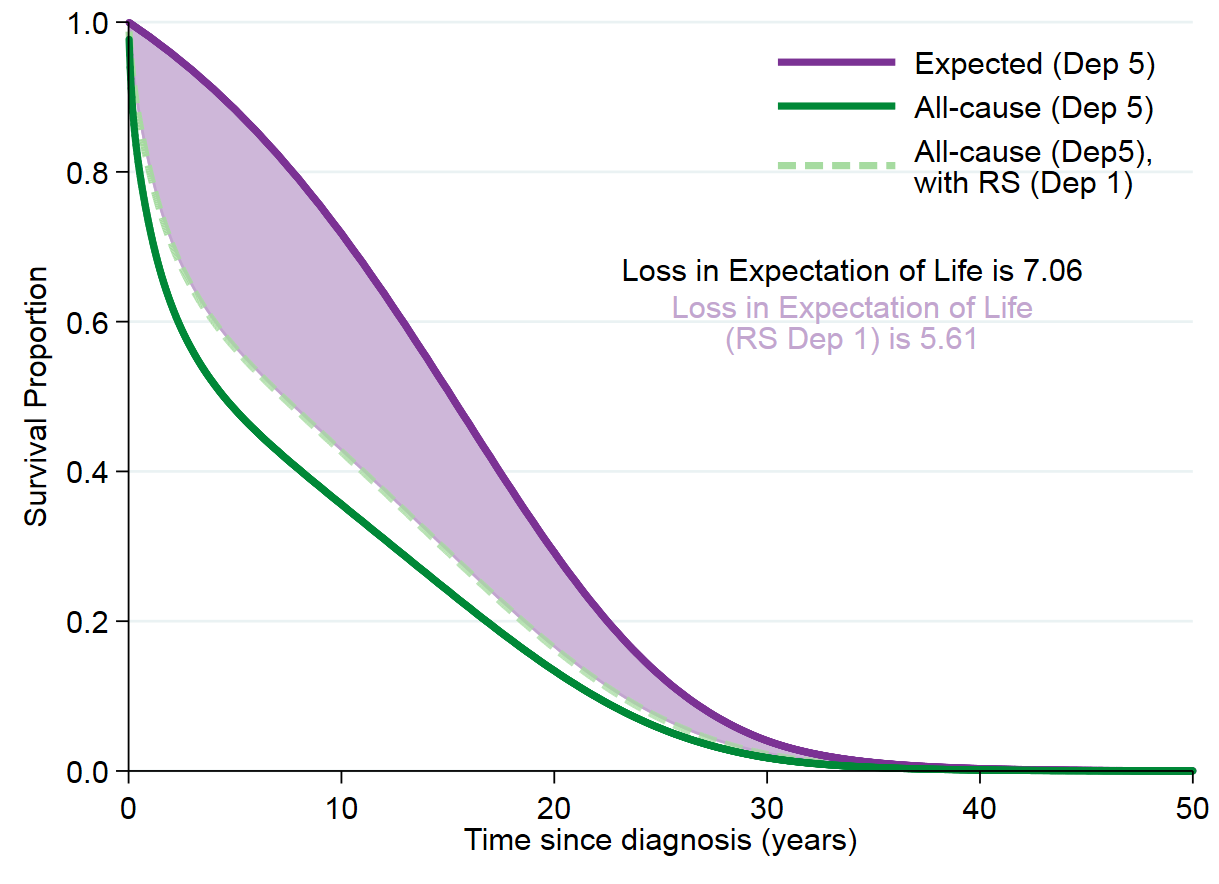


B


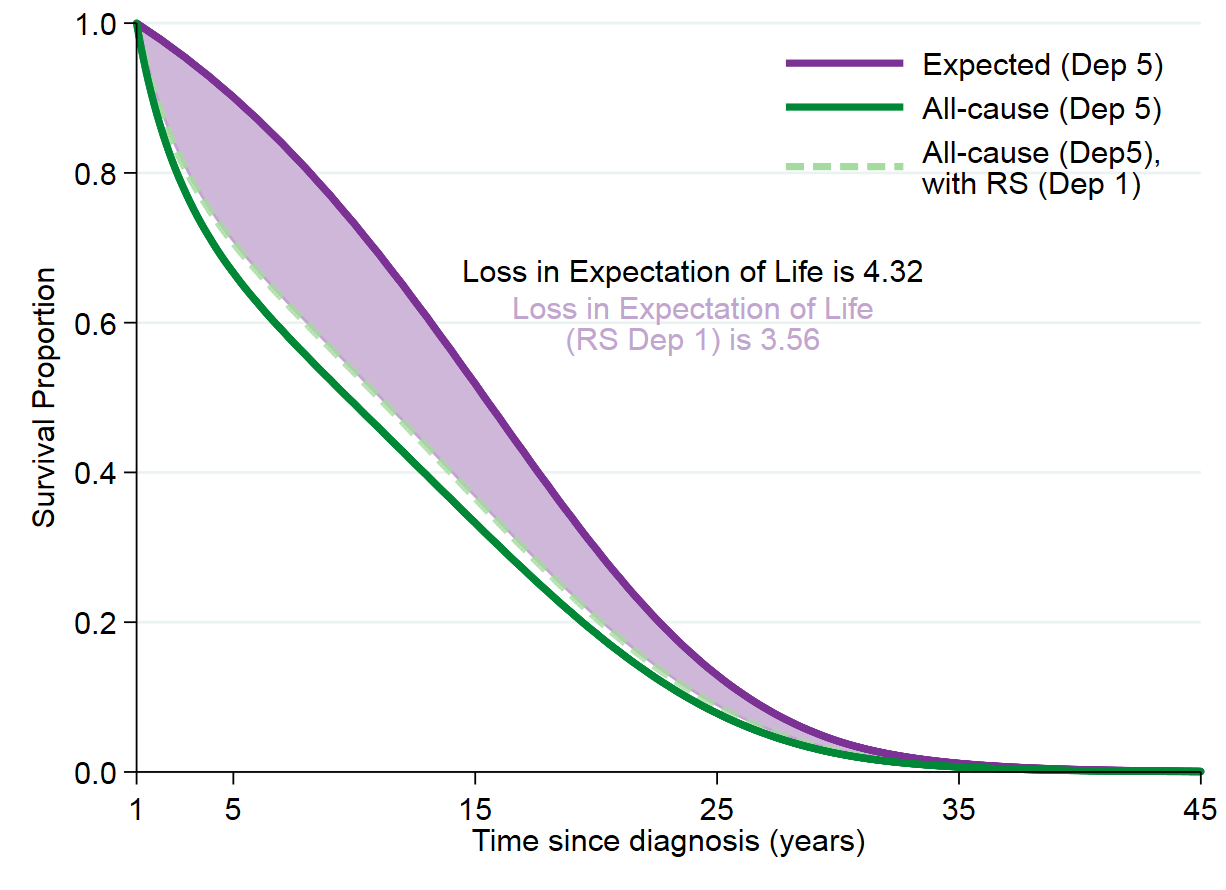

Supplement: Supplementary file 1 — Supplemental material [file 41416_2019_455_MOESM1_ESM.docx]
